# Assessing the adherence and acceptability to iron and folic acid compared with multiple micronutrient supplements during pregnancy: a cluster-randomized noninferiority trial in Cambodia

**DOI:** 10.1016/j.ajcnut.2025.04.033

**Published:** 2025-05-05

**Authors:** Cassandra Sauer, Mai-Anh Hoang, Hou Kroeun, Aman Sen Gupta, Rem Ngik, Meng Sokchea, Jocelyne M Labonté, Mary Chea, Rolf Klemm, Ashutosh Mishra, Aishwarya Panicker, Vin Sokhal, Crystal D Karakochuk

**Affiliations:** 1 Food, Nutrition & Health, The University of British Columbia, Vancouver, BC, Canada; 2 Healthy Starts, BC Children’s Hospital Research Institute, Vancouver, BC, Canada; 3 Helen Keller International, New York, NY, United States; 4 Helen Keller International, Phnom Penh, Cambodia; 5 Helen Keller International, Lalitpur, Nepal; 6 Interdisciplinary School of Health Sciences, University of Ottawa, Ottawa, Canada; 7 National Nutrition Program, Ministry of Health, Phnom Penh, Cambodia; 8 International Health, John Hopkins Bloomberg School of Public Health, Baltimore, MD, United States; 9 Vitamin Angel Alliance, Santa Barbara, CA, United States

**Keywords:** pregnancy, global health, policy, prenatal supplements, nutrition, iron and folic acid, multiple micronutrient supplements, Cambodia

## Abstract

**Background:**

The Cambodian Ministry of Health is exploring transitioning from iron and folic acid (IFA) to multiple micronutrient supplements (MMS) during pregnancy and is seeking rigorous evidence to inform this policy change.

**Objective:**

We aimed to assess the adherence and acceptability of MMS compared with IFA supplementation during pregnancy.

**Methods:**

We conducted an open-label cluster-randomized noninferiority trial across 48 health centers in Cambodia. A total of 1546 healthy pregnant individuals (18–45 y) were recruited at their first antenatal care (ANC) visit (<14 weeks of gestation) and randomized to 1 of 3 arms at the health center level: *1*) IFA for 90 d (IFA-90, *n* = 515), the current standard of care; *2*) MMS for 180 d via 1 180-tablet bottle (MMS-180, *n* = 516); or 3) MMS for 180 d via 2 90-tablet bottles (MMS-90, *n* = 515). Our primary outcome was the noninferiority of adherence rates of MMS-180 compared with IFA-90, assessed by tablet counts and compared against a predefined noninferiority margin of −15%. Mixed-effects linear regression models were used to estimate the mean difference (95% confidence interval [95% CI]) in adherence rates. Our secondary outcomes included the mean difference in ANC attendance between the MMS groups and the acceptability of MMS across 6 domains.

**Results:**

Overall, 88% of participants completed the trial, with high mean adherence rates across arms (91% for IFA-90, 95% for MMS-180, and 95% for MMS-90). The adjusted mean (95% CI) difference in adherence rates between MMS-180 and IFA-90 groups was 3.9% (1.7, 6.2). The adjusted mean (95% CI) difference in ANC visits for MMS-180 and MMS-90 groups was 0.0 (−0.1, 0.2) visits. The acceptability of MMS was positive (90%–100% “agreement” across 6 domains).

**Conclusions:**

Both IFA and MMS were highly acceptable, yet adherence to MMS was superior to IFA. These findings support the transition from IFA to MMS in Cambodia.

This trial was registered at Clinicaltrials.gov as NCT05867836.

## Introduction

Current guidelines in Cambodia recommend the consumption of iron and folic acid (IFA) supplements during pregnancy for 90 d, starting as early as possible [1]. However, there has been a global push based on evidence showing its superiority to replace IFA with multiple micronutrient supplements (MMS), which include IFA and 13 other vitamins and minerals [2,3]. A 2019 review concluded that prenatal supplementation with MMS reduced risk of low birth weight by 12% and risk of small for gestational age by 8%, compared with IFA [4]. A 2017 independent patient meta-analysis found similar results to the 2019 review, in addition to confirming that MMS provided greater beneficial effects among individuals with anemia [5]. Based on this evidence, the WHO updated its antenatal care (ANC) guidelines in 2020 to recommend MMS in the context of “rigorous research,” such as acceptability and cost-effectiveness trials [6].

Although global studies have shown the positive outcomes associated with MMS as compared with IFA, there is limited evidence of its use in the Cambodian context. After conducting a landscape analysis and initiating consultations with key stakeholders, the Cambodian Ministry of Health (MoH) expressed a strong interest to transition from IFA to MMS during pregnancy but sought rigorous evidence in the Cambodian context to inform this policy change.

The adherence and acceptability of MMS may differ from IFA, particularly because the daily dose of elemental iron in IFA (60 mg) is double that of MMS (30 mg). The dose of iron in an oral supplement can influence tolerability (e.g., gastrointestinal side effects) and the organoleptic properties (e.g., smell, taste, appearance) of the supplements, which could ultimately influence acceptability and adherence. There was also a need to assess whether pregnant individuals would adhere to a 180-d supplementation regimen of MMS compared with the standard of care in Cambodia (90-d regimen of IFA), and whether the quantity of MMS tablets provided at 1 visit (i.e., 90 vs. 180 tablets) influenced MMS adherence and ANC attendance. To address these gaps, our primary objective was to determine if MMS adherence rates were noninferior to those of IFA. Our secondary objective was to determine if the distribution of MMS in 1 bottle (180 tablets) compared with 2 bottles (90 tablets each) influenced adherence or ANC attendance. In addition, we also aimed to assess the acceptability of MMS and IFA among pregnant individuals across multiple domains, including assessment of the organoleptic properties of the tablets, packaging, burden of consumption, perceived effectiveness, opportunity cost, and self-efficacy. This study provides the necessary evidence to inform the Cambodia MoH’s decision on the transition from IFA to MMS, potentially leading to improved maternal and child health outcomes in Cambodia.

## Methods

### Study design

This study was an open-label cluster-randomized, 3-arm parallel, noninferiority trial conducted between March and December 2023. The study took place in the centrally located, semirural province of Kampong Thom, Cambodia. The trial was registered with clinicaltrials.gov (NCT05867836), and the protocol has been published [7]. Ethical approval was received from the University of British Columbia Clinical Research Ethics Board (H23-01316) and the National Ethics Committee for Health Research in Cambodia (number: 056). This study was conducted in accordance with the principles outlined in the Declaration of Helsinki. Written informed consent was obtained from all participants upon study enrollment.

Cluster randomization at the health center level was employed for practical reasons of logistical ease and to prevent contamination across trial arms related to potential preferences of pregnant individuals or health care providers for a specific intervention. Health centers included in the study were in the operational districts of Kampong Thom, Baray Santuk, and Staung, all within Kampong Thom province. Forty-eight of 56 health centers in Kampong Thom province were randomly selected for study inclusion in proportionate amounts across the 3 operational districts, based on the required sample size and anticipated recruitment time. No health centers refused to participate in the study.

### Participants

Healthy pregnant individuals (18–45 y), in their first 14 wk of a low-risk pregnancy (defined as a singleton pregnancy with the absence of any medical condition), living in Kampong Thom province, and willing to have data collectors conduct home visits were eligible for inclusion. Exclusion criteria included plans to relocate outside Kampong Thom within 6 mo and involvement in other nutrition programs beyond the standard of government care.

### Randomization

Forty-eight health centers were randomly assigned to 1 of the 3 arms using a computer-generated list in Excel, with a total of 16 health centers per arm. Study participants were not blinded to their treatment arm and, thus, were aware if they were receiving IFA supplements or MMS. Our goal was to provide IFA as per the current standard of care in Cambodia, which was providing the tablets in small, resealable, and transparent bags. This packaging differed from that of MMS, thereby removing the possibility of blinded interventions. A total of 1546 healthy pregnant individuals were recruited at their first ANC visit and randomly assigned to 1 of 3 arms at the health center level: *1*) IFA for 90 d (IFA-90), the current standard of care; *2*) MMS for 180 d via 1 180-tablet bottle (MMS-180); or *3*) MMS for 180 d via 2 90-tablet bottles (MMS-90).

The IFA tablets contained 60 mg elemental iron (as ferrous fumarate) and 400 μg folic acid [1]. The IFA tablets were provided in kind by the Cambodian MoH and manufactured by Medicamen Biotech Ltd. The MMS tablets were provided in kind by Kirk Humanitarian and manufactured by Contract Pharmacal Corp. The MMS tablets were based on the formulation of the UNIMMAP MMS, which contain 15 micronutrients: 30 mg elemental iron (as ferrous fumarate), 400 μg folic acid, 800 μg vitamin A, 200 IU vitamin D, 10 mg vitamin E, 70 mg vitamin C, 1.4 mg thiamin, 1.4 mg riboflavin, 18 mg niacin, 1.9 mg vitamin B6, 2.6 μg vitamin B12, 2 mg copper, 150 μg iodine, 65 μg selenium, and 15 mg zinc [8]. Both IFA and MMS are included in the 2023 WHO Model List of Essential Medicines, proving safety and efficacy for public health interventions in the intended population [9].

### Procedures

Recruitment at health centers began in March 2023, and enrollment continued for 4 mo, with trial completion in December 2023. Health care providers approached pregnant individuals for their interest in participating during their first ANC visit. Participants enrolled in the IFA-90 arm received 90 tablets in total: 60 tablets at their first ANC visit (<14 weeks of gestation) and 30 tablets at their second ANC visit (∼20–24 weeks of gestation), as per MoH guidelines. Participants enrolled in the MMS-90 arm received 180 tablets in total: 90 tablets in 1 bottle at their first ANC visit and 90 tablets in a second bottle at their second ANC visit. Participants enrolled in the MMS-180 arm received 180 tablets in total: all 180 tablets in 1 bottle at their first ANC visit.

All participants received the same prenatal counseling by trained health care workers, as per the current standard of care in Cambodia, for example: “Supplements are good for the health of the mother and infant. They help the infant be strong and smart.” Additionally, participants received educational messaging in the form of printed handouts or packaging labels, which included key health messages and instructions on how to take the supplements, for example: “Take every night before going to bed.”

Helen Keller International hired and trained 12 experienced Cambodian staff as the data collectors for the study. Data collectors spoke the local language and had previous experience in research data collection and travel to remote areas in Cambodia. Before study commencement, the data collectors completed comprehensive training on the study’s protocol, standard operating procedures, ethical considerations, questionnaire administration, and tablet counting. Quality assurance measures included regular field supervision, weekly team meetings, and periodic data audits to maintain high standards of data integrity throughout the study.

### Data collection

Sociodemographic data were collected in the local language by research staff using interviewer-administered questionnaires that collected data on the participant’s age, ethnicity, religion, educational level, marital status, and gravida. All data were obtained by self-report at the participant’s household. Adherence was assessed by tablet counts: for the IFA group, data collectors counted their remaining tablets at the 30- and 90-d time points. For the MMS groups, data collectors counted their remaining tablets at the 90- and 180-d time points (tablets were not counted in the MMS groups at the 30-d time point). IFA tablets were counted inside their original transparent packaging. MMS tablets were poured into and counted inside a clean transparent plastic bag. Duplicate counts were taken; if a discrepancy was observed, a third count was completed. Acceptability data were collected using oral interviewer-administered quantitative questionnaires that asked participants to rate their level of agreement or disagreement with each of the statements using a 5-point Likert scale. Some examples of the statements included in the questionnaire include “I like the taste,” “The package gives me confidence that the supplements are from a quality manufacturer,” and “The supplement is good for my baby's health.” The Likert scale included the potential responses of “strongly disagree,” “disagree,” “neutral,” “agree,” or “strongly agree” and provided participants with corresponding emoticons for each response. Participants were asked to point to the emoticon that aligned with their response for each statement. Participants were also asked if they experienced positive (e.g., feeling healthier or increased energy) and negative effects (e.g., nausea or constipation) from the supplement based on a preset list of options. Participants were informed upon study enrollment that home visits would be scheduled to collect the adherence and acceptability data. Participants were also reminded again 7 d before the intended visit.

### Statistical analysis

Sample size estimations were calculated using adherence (%) as both a binary and a continuous outcome. We chose the more conservative sample size calculation for adherence as a binary outcome to ensure we were powered for both outcomes. In consultation with a biostatistician and using an online calculator tool (Sealed Envelope Ltd.), the sample size estimated was determined using a formula that takes into account the desired level of confidence (*α* = 0.025), power (*β* = 0.1), and the expected true adherence in the standard (76%; IFA) and experimental (74%; MMS) treatment group, as well as the minimum detectable difference between the success rates (−15%) [10]. This noninferiority margin was selected in consultation with maternal and child health experts and in consideration of the clinical significance of adherence outcomes. A design effect of 2 was selected to account for the clustering within health centers and the 3 arms of the trial. Based on this calculation, 468 participants were required in each group to detect a noninferiority parameter of −15% adherence between MMS-180 and IFA-90. To allow for a 10% loss to follow-up, a total of 515 participants per arm were required, for a total sample size of 1545 for the trial.

#### Primary outcome

Our primary outcome was the noninferiority of adherence rates of MMS-180 compared with IFA-90, assessed by tablet counts and compared against a predefined noninferiority margin of −15%. Noninferiority can be claimed if the mean difference and 2-sided 95% CI are fully to the right of the noninferiority margin. Multilevel mixed-effects generalized linear regression models were used to estimate the mean difference (95% CI) in adherence rates, controlling for health centers and applying a Bonferroni correction (for the 3 arms). The primary analysis was performed on an “intention-to-treat” basis, where all participants were analyzed according to their allocated intervention group. No imputation of missing data was performed. Analyses were completed using Stata/IC version 18.0 (Stata Corp LLC).

#### Secondary outcomes

Multilevel mixed-effects generalized linear regression models were also used to estimate the mean difference (95% CI) in ANC attendance using a Poisson distribution, controlling for health centers (clusters) and applying a Bonferroni correction. Only the MMS groups were included as the purpose of this analysis was to determine if providing all 180 MMS tablets via 1 bottle at ANC 1 compared with providing 180 tablets via 2 bottles at ANC 1 and ANC 2 had any impact on ANC attendance.

Acceptability was assessed using a quantitative questionnaire based on a published framework for acceptability of health care interventions [11]. The 6 domains in the questionnaire include physical properties of the supplement, packaging, burden to take, perceived effectiveness, opportunity cost, and self-efficacy. Participant responses were obtained after ∼90 d of the intervention. Agreement was calculated by totaling the responses in the “strongly agree” and “agree” categories, whereas disagreement was calculated by totaling the responses in the “strongly disagree” and “disagree” categories.

## Results

### Participant characteristics

**FIGURE 1:**
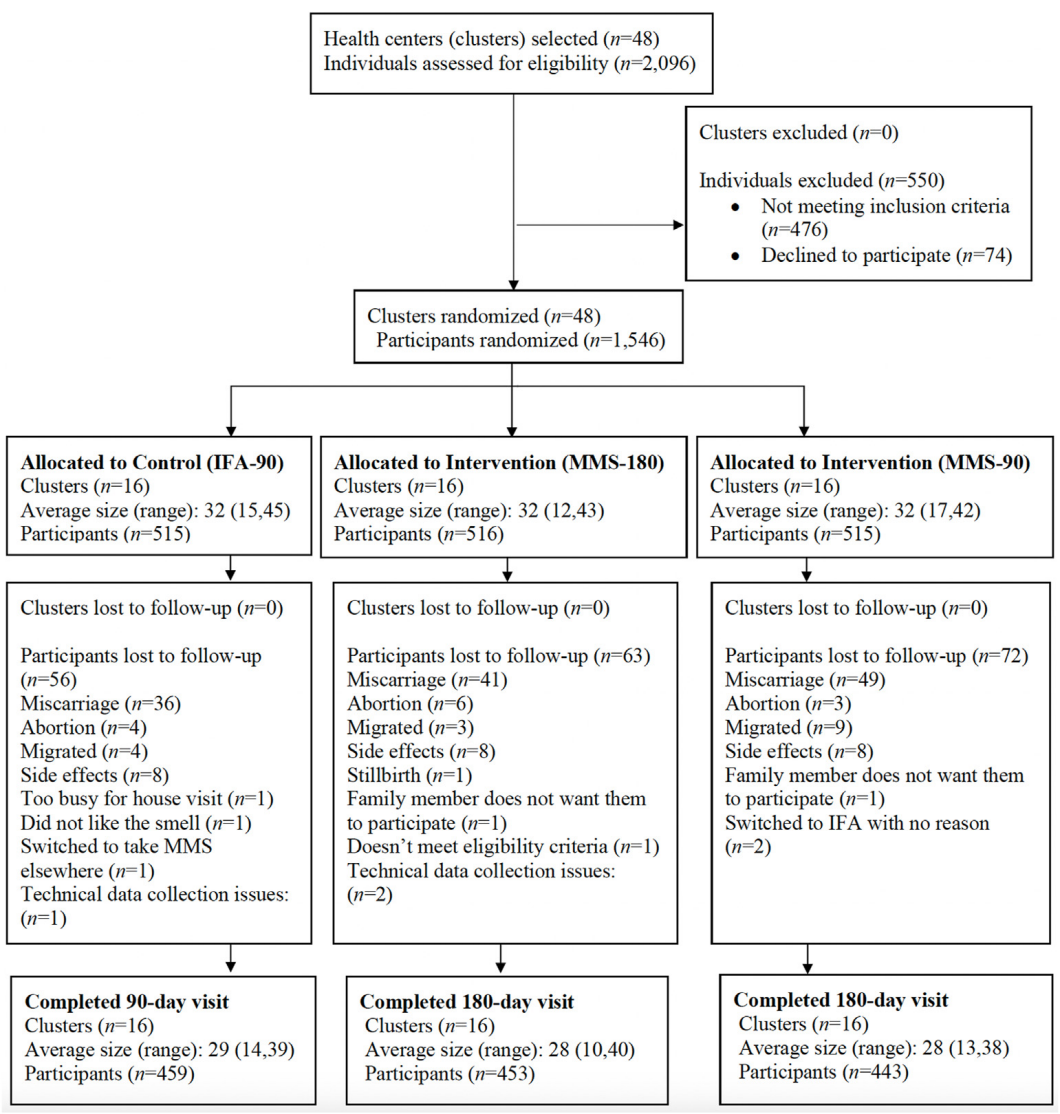
Participant flow diagram. IFA, iron and folic acid; IFA-90, IFA for 90 d; MMS, multiple micronutrient supplements; MMS-180, MMS for 180 d via 1 180-tablet bottle; MMS-90, MMS for 180 d via 2 90-tablet bottles.

Between March 1 and June 23, 2023, a total of 2096 pregnant individuals were screened in 48 health centers. Of these, 74 declined to participate and 476 did not meet the eligibility criteria, including having a gestational age > 14 wk (*n* = 254) and not planning to live in Kampong Thom province for the next 6 mo (*n* = 148). The remaining 1546 individuals were enrolled and 1355 (88%) completed the trial (Figure 1). Miscarriage was the most common reason for discontinuing participation in the trial, with the rates of miscarriage similar (7%, 8%, 10%, chi-square test, *P* = 0.327) across all 3 arms. Overall, enrolled participants had a mean ± SD age of 28 ± 6 y, gestational age of 8 ± 3 wk, 99% were married, 94% completed primary school, 70% had been previously pregnant, and 60% were in the second highest or highest quintile for the household asset equity score (Table 1) [12].

**TABLE 1:** Demographic characteristics of enrolled participants by trial arm^1^.

Characteristic	IFA-90 (*n* = 515)	MMS-180 (*n* = 516)	MMS-90 (*n* = 515)
Age, year, mean ± SD	28±6	28±6	28±6
Marital status, married, *n* (%)	508 (99)	503 (98)	509 (99)
Gravidity, *n* (%)			
Primigravida	134 (26)	179 (35)	155 (30)
Multigravida	381 (74)	337 (65)	360 (70)
Multigravida, number of pregnancies, mean ± SD	2±1	2±1	2±1
Gestational age at first ANC visit, weeks, mean ± SD	8±3	8±3	8±2
Education completed, *n* (%)			
None	25 (5)	23 (4)	45 (9)
Primary	220 (43)	208 (40)	193 (38)
Secondary	147 (29)	181 (35)	168 (33)
Higher secondary	89 (17)	72 (14)	76 (15)
University and above	33 (6)	30 (6)	31 (6)
Nonformal education	0 (0)	1 (0)	1 (0)
Household asset equity score^2^, *n* (%)			
Lowest	6 (1)	5 (1)	4 (1)
Second Lowest	46 (9)	39 (8)	35 (7)
Middle	166 (32)	142 (28)	173 (34)
Second Highest	139 (27)	178 (35)	139 (27)
Highest	157 (31)	151 (29)	162 (32)

Abbreviations: ANC, antenatal care; IFA, iron and folic acid; IFA-90, IFA for 90 d; MMS, multiple micronutrient supplements; MMS-180, MMS for 180 d via 1 180-tablet bottle; MMS-90, MMS for 180 d via 2 90-tablet bottles; SD, standard deviation.

1 Total *n* = 1546.

2 The household asset equity score is based on the 2014 Cambodia Demographic and Health Survey [12] as an indicator of the relative level of wealth that is used as a proxy for expenditure and income measures.

### Adherence

**FIGURE 2:**
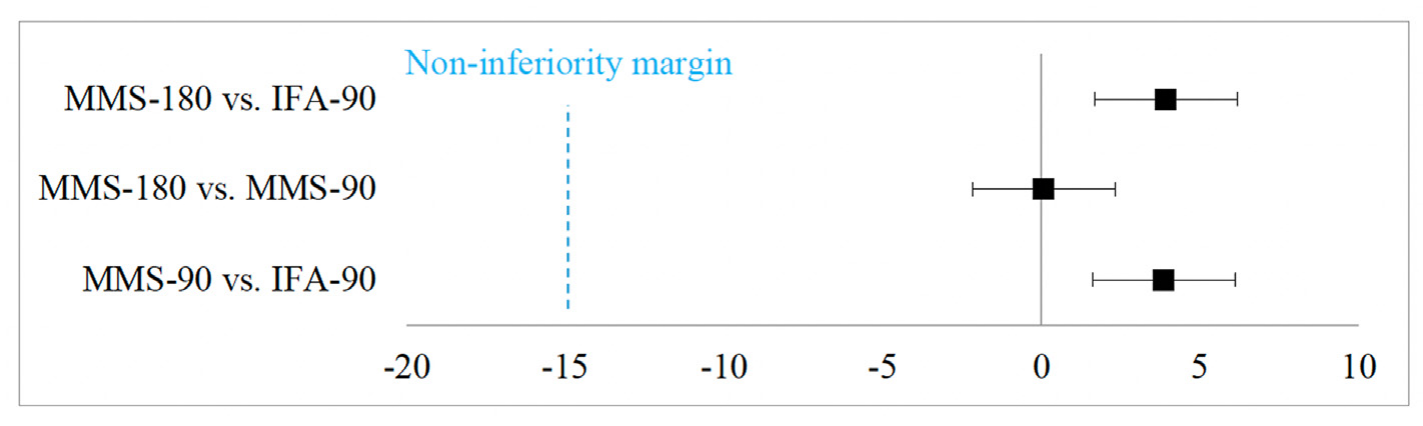
Noninferiority of adherence rates. Adjusted mean difference (95% CI) in adherence rates between the intervention arms (MMS minus IFA). The primary outcome (MMS-180 vs. IFA-90) shows that adherence to MMS-180 is “superior” to adherence to IFA-90 as the CI lies fully to the right of zero [13]. CI, confidence interval; IFA, iron and folic acid; IFA-90, IFA for 90 d; MMS, multiple micronutrient supplements; MMS-180, MMS for 180 d via 1 180-tablet bottle; MMS-90, MMS for 180 d via 2 90-tablet bottles.

Overall, mean (95% CI) adherence rates were high across all groups at the study end point (Table 2); results from the first and second visits are shown in Supplemental Table 1. Adherence was higher in both MMS groups as compared with IFA-90 (*P* < 0.001). Thus, in this circumstance, the noninferiority analysis of adherence rates to MMS-180 (180 d) compared with IFA-90 (90 d) is declared as “noninferior” [13], as the CI (1.7%, 6.2%) lies to the right of the “a priori” defined noninferiority margin of −15% (Figure 2) [13]. In fact, because the range of the CI falls completely to the right of 0, adherence to MMS-180 is declared as “superior” to adherence to IFA-90 [13]. Sensitivity analyses were conducted to assess the robustness of the findings including adjusting for potential confounders such as participant characteristics (age, education, household asset equity score, and gestational age at first ANC visit) (Supplemental Table 2). The results of these sensitivity analyses were not statistically different from those of the primary analysis, confirming the robustness of our findings.

**TABLE 2:** Adherence and ANC attendance outcomes^1^.

	IFA-90 *n* = 459	MMS-180 *n* = 453	MMS-90 *n* = 443	MMS-180 vs. IFA-90 comparison^2^	
	(90-d)	(180-d)	(180-d)	Mean difference (95% CI)	*P* value
Adherence rate					
Unadjusted rate, mean (95% CI)	91% (90, 92)	95% (94, 96)	95% (94, 95)	—	—
Adjusted rate (model), predicted marginal mean (95% CI)	91% (89, 92)^a^	95% (93, 96)^b^	94% (93, 96)^b^	3.9 (1.7, 6.2)	*P*<0.001
ANC visit attendance^3^				MMS-180 vs. MMS-90 (180-day time point) comparison^2^	
Unadjusted mean (95% CI)	2.6 (2.5, 2.6)	5.0 (4.9, 5.1)	5.0 (4.8, 5.1)	—	—
Adjusted model, predicted marginal mean (95% CI)	2.6 (2.4, 2.7)	5.0 (4.6, 5.4)^a^	5.0 (4.5, 5.4)^a^	0.0 (−0.1, 0.2)	*P*=1.000

Abbreviations: ANC, antenatal care; CI, confidence interval; IFA, iron and folic acid; IFA-90, IFA for 90 d; MMS, multiple micronutrient supplements; MMS-180, MMS for 180 d via 1 180-tablet bottle; MMS-90, MMS for 180 d via 2 90-tablet bottles.

1 Total *n* = 1355. All values are mean or marginal means (95% CI).

2 A generalized linear mixed-effects model was used to predict the marginal mean (95% CI) for each group accounting for health center clusters. Marginal mean values with the same superscript letter in a row are not statistically different across intervention groups (*P* < 0.05; Bonferroni-adjusted for multiple comparisons).

3 IFA-90 ANC attendance was only reported at the 90-d time point (as this was their last point of data collection).

### ANC attendance

The mean ± SD ANC visits attended for MMS-180 and MMS-90 were 5 ± 1.5 and 5 ± 1.4 visits, respectively (Table 2). The adjusted mean (95% CI) number of ANC visits attended for MMS-180 and MMS-90 was 5.0 (4.6, 5.4) and 5.0 (4.5, 5.4) visits, respectively, with an adjusted mean (95% CI) difference of 0.0 (−0.1, 0.2) visits.

### Acceptability

**FIGURE 3:**
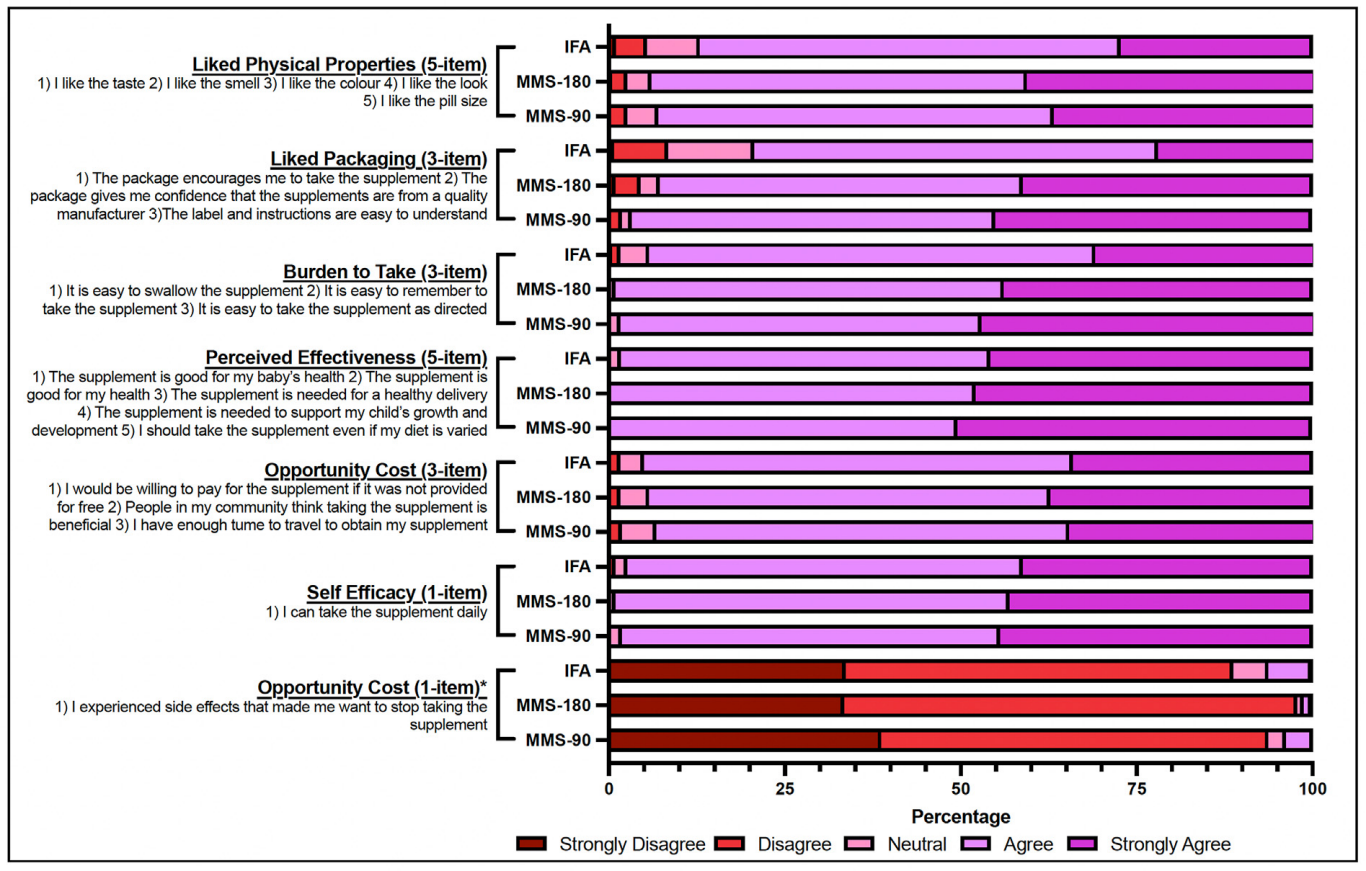
Domain-specific acceptability of MMS and IFA. IFA, iron and folic acid; IFA-90, IFA for 90 d; MMS, multiple micronutrient supplements; MMS-180, MMS for 180 d via 1 180-tablet bottle; MMS-90, MMS for 180 d via 2 90-tablet bottles. ∗Due to the nature of this statement (I experienced side effects that made me want to stop taking the supplement), it was removed from the main opportunity cost domain group as the positive response was the inverse of the other statements in the domain.

The acceptability of MMS was overwhelmingly positive ranging from 90% to 100% “agreement” across the domain-specific groups (Figure 3). In terms of the physical properties, 92% of participants in the MMS groups liked the supplement’s taste, smell, color, look, and size, compared with 87% in the IFA group. The main property of disagreement and neutrality for all groups was “I like the smell” and “I like the taste.” Overall, MMS still showed more agreeable physical properties than IFA. Packaging acceptability was higher in the MMS groups (94%) compared with the IFA group (79%). The burden to take the supplement was lower in the MMS groups (99%) than in the IFA group (94%). All study groups had >95% agreement toward the perceived effectiveness of the supplement and >90% agreement toward the opportunity cost of the supplement. There was positive agreement (>95%) across all groups with respect to self-efficacy to take the supplement. Finally, when shown the statement “I experienced side effects that made me want to stop taking the supplement,” responses were similar across groups with >85% in disagreement with the statement.

### Perceived adverse side effects

At the 30-d time point, 36% (*n* = 497/1400) of participants reported adverse side effects in the past month; the proportion of participants who reported any perceived adverse side effects differed by intervention group (IFA-90: 48%, MMS-180: 23%, MMS-90: 36%; chi-square, *P* < 0.001). Of the 497 participants who reported any negative side effects, 68% (*n* = 337/497; IFA-90: 72%, MMS-180: 62%, MMS-90: 67%; chi-square, *P* = 0.052) reported nausea or vomiting, 32% (*n* = 158/497; IFA-90: 38%, MMS-180: 28%, MMS-90: 26%; chi-square, *P* = 0.018) reported tiredness or fatigue and 32% (*n* = 157/497; IFA-90: 33%, MMS-180: 37%, MMS-90: 26%; chi-square, *P* = 0.10) reported stomach cramping. Although less frequently reported, there were significantly less adverse effects of constipation (*n* = 108/497; IFA-90: 29%, MMS-180: 15%, MMS-90: 15%; chi-square, *P* < 0.001) and heartburn (*n* = 45/497; IFA-90: 14%, MMS-180: 4%, MMS-90: 5%; chi-square, *P* = 0.002) in the MMS groups.

At the 90-d time point, 17% (*n* = 227/1340) of participants reported adverse side effects in the past month; the proportion of participants who reported any perceived adverse side effects differed by intervention group (IFA-90: 27%, MMS-180: 9%, MMS-90: 14%; chi-square test, *P* < 0.001). Of the 227 participants who reported any negative side effects, 55% (*n* = 125/227) reported nausea or vomiting, 33% (*n* = 75/227) reported stomach cramping, and 26% (*n* = 59/227) reported tiredness or fatigue. There were significant differences by intervention groups of those who experienced stomach cramping (chi-square test, *P* = 0.008) and those who experienced tiredness (chi-square test, *P* = 0.008).

At the 180-d time point (data only collected for MMS groups), only 4% (*n* = 36/898) of participants reported adverse side effects in the prior month. Of those 36 participants who reported any negative side effects, 42% (*n* = 15/36) reported other reasons such as sickness from the smell of the tablets and dizziness and 33% (*n* = 12/36) reported nausea or vomiting.

### Perceived benefits experienced

At the 90-d time point, 91% (*n* = 1215/1340) of participants reported positive experiences from their allocated supplement with the IFA group reporting significantly fewer positive experiences compared with those in the MMS groups (IFA-90: 78%, MMS-180: 98%, MMS-90: 97%; chi-square, *P* < 0.001). Of the benefits experienced most frequently, 81% (*n* = 984/1215) reported having increased energy, 73% (*n* = 892/1215) reported having improved sleep, 72% (*n* = 878/1215) reported feeling healthier, and 70% (*n* = 850/1215) reported feeling happier.

At the 180-d time point (data only collected for MMS groups), 99% (*n* = 887/898) of participants reported positive experiences from their allocated supplement. Of the benefits experienced most frequently, 87% (*n* = 772/887) reported having increased energy, 87% (*n* = 768/887) reported having improved sleep, and 85% (*n* = 756/887) reported feeling happier.

## Discussion

In this cluster-randomized trial in Cambodia, we found that adherence rates in the IFA (∼91%) and MMS groups were high (∼95%) and, in fact, adherence to MMS was superior to IFA. We acknowledge that adherence, as assessed in clinical research, may be inflated due to the increased participant follow-up and the awareness of being monitored [14]. However, we infer 2 important conclusions here. First, the transition to MMS from IFA during pregnancy is unlikely to decrease adherence and may in fact increase adherence in Cambodia. Second, even with extending the duration of MMS supplementation from 90 to 180 d, we are confident that high adherence can be sustained throughout the longer period.

Over the last 20 y, adherence to IFA during pregnancy has drastically improved in Cambodia. In 2000, the Cambodia Demographic and Health Survey (DHS) found only 2% of pregnant individuals reported taking a minimum of 90 tablets of IFA during pregnancy, as compared with 76% in 2014 and 88% in 2021 [1,14,15]. This is likely due to evolving government strategies that aim to improve training for midwives and the quality of ANC services [16]. We speculate that this has led to an increase in attendance to ANC services and understanding of the importance of nutritional supplementation during pregnancy. This was also evidenced by our qualitative research in Kampong Thom, which found that individual knowledge on prenatal supplementation, access and quality of ANC, and their perceptions and beliefs about MMS were all contributing factors to MMS adherence [17]. Trials in other countries have also found MMS adherence to be high. A trial in Mali among pregnant individuals (*n* = 70) found that the mean number of tablets consumed over 9 mo was higher in the MMS than in the IFA group (95% vs. 92%; *P* = 0.008) [18]. Adherence to both MMS and IFA was also found to be high (∼85%) in a trial in Indonesia (*n* = 31,290), where pregnant individuals’ preferences of the supplement appearance and packaging was considered for the design [19].

Our findings confirmed that adherence rates and ANC attendance did not differ whether MMS was distributed via 1 180-tablet bottle or 2 90-tablet bottles. Current Cambodian guidelines suggest that pregnant individuals attend ≥4 ANC visits during pregnancy [20]. Participants in our trial attended a mean number of a mean number of 5 visits, regardless of how many MMS tablets they received. This finding is consistent with 2021 DHS data, which reported that ∼86% of pregnant individuals had ≥4 ANC visits during their last pregnancy and 28% had 8 or more ANC visits [15]. In summary, our trial results suggest that there is flexibility in packaging options for MMS distribution.

Packaging type (e.g., blister packs or bottle sizes) and other factors, such as shelf-life, cost, and accessibility, should also be considered when determining the distribution mechanism for MMS. In Indonesia, health care providers favored blister packs due to safety, ease of adherence monitoring, and concerns about reduced adherence and product degradation over time if too many pills were provided at once [21]. In contrast, pregnant individuals in Indonesia preferred bottles, perceiving them as higher quality because they resembled more expensive commercial products [21]. In Bangladesh, pregnant individuals preferred blister packs over bottles as they were perceived as easy to dispense and keep track of daily intake [22].

We assessed participant acceptability to MMS across 6 domain-specific groups, based on a published framework for acceptability of health care interventions [11]. Overall, acceptance of MMS was very high, and ultimately the less-acceptable factors (smell and taste) did not appear to negatively influence adherence (∼95%). Potential ways to mitigate the “smell” of the supplement would be to apply a thicker film coating to try and mask the smell or include ingredients to improve the smell and/or taste (e.g., flavor) [23]. However, these modifications tend to increase cost and must also be considered for cultural appropriateness. We believe that the understanding of the importance of supplementation likely led participants to continue to consume the supplements regardless of any unpleasant smell, taste, or side effects. This was also confirmed in the “opportunity cost domain” when participants were asked if they experienced side effects that made them want to stop taking the supplement. Participants had the highest disagreement on their willingness to purchase the supplement. A study in Burkina Faso found that the cost of the supplement can be a barrier as many expect MMS to be free [24]. However, in the Philippines and Bangladesh, there was a general willingness to pay for the supplement and the desire for MMS to be available in pharmacies in addition to health centers [25,26].

Overall, we observed low rates of perceived adverse side effects across all arms, with the MMS groups reporting fewer perceived adverse side effects compared with the IFA group. The most common negative side effects reported (stomach cramping, nausea, and tiredness) are also inherently common during pregnancy. Our observation that a higher proportion of adverse side effects were reported at the 30-d time point (∼35%) compared with the 90-d time point (∼17%) and even fewer at the 180-d time point (∼4%) suggests that likely the adverse side effects reported are more likely pregnancy related than a result of the supplement. However, we did observe a higher proportion of adverse side effects in the IFA group (27%) than in the MMS groups (MMS-180: 9%, MMS-90: 14%). These observed differences may be attributed to several factors. First, the lower iron dose of elemental iron in MMS (30 mg) compared with IFA (60 mg) likely contributed to the reduced incidence of gastrointestinal side effects, such as nausea, vomiting, and stomach cramping [27]. This could also explain the higher adherence rates observed in the MMS groups. It is important to note that some participants (*n* = 24, 8 per arm) who experienced side effects early on dropped out prior to study completion and would not be captured in the overall perceived adverse effect estimate. However, we observed very high perceived “benefits” experienced across both the MMS and IFA groups. This has also been reported in Mali, where pregnant individuals perceived IFA and MMS as very acceptable in terms of physical properties and benefits for the mother and infant [18].

Strengths of this study include the appropriately powered sample size (*n =* 1546) with recruitment from 48 different health centers (clusters), representing ∼86% of all health centers in Kampong Thom. However, we recognize that our findings may not be generalizable to provinces outside Kampong Thom. Several factors may influence adherence and ANC attendance, such as accessibility and distance to health centers, customer service, and the influence of family members and health care providers, which may vary across provinces in Cambodia. Recruitment from health centers inherently limits our ability to reach the most vulnerable females, who may not be able to access the health center. Although the study design and implementation were robust, the unblinded nature of the trial could have introduced performance and detection bias. Additionally, the presence of data collectors conducting home visits to count tablets, could have influenced behaviors, although this was equally applicable to all 3 trial arms, it could have influenced our adherence results but was unlikely to impact our primary trial outcome.

Globally, there has been a push to replace IFA with MMS, based on evidence showing its superiority [2,3]. Formative research is often the first step, preceding a national policy change, which can include a landscape analysis, stakeholder consensus building, or pilot testing [28]. Cambodia is currently in the implementation phase, along with many other countries [29]. Our findings suggest that the high adherence rates and acceptability of MMS support the transition from IFA to MMS in Cambodia. Next steps should include an MMS supply chain assessment and development of optimal delivery strategies for timely distribution. Our study results provide important considerations and lessons learned for stakeholders in Cambodia and other countries considering a transition from IFA to MMS.

## Author contributions

The authors’ responsibilities were as follows – MH, RK, AG, RN, AM, AP: designed the trial; CS, MH, HK, AG, RN, MS: conducted research; HK, MC, AM, AP, VS: provided essential materials; CS, AG, CDK: conducted the statistical analyses; CS, MH, RK, JML, CDK: wrote paper; CS: had primary responsibility for final content; and all authors read and approved the final manuscript.

## Data availability

Data described in the manuscript, code book, and analytic code will be made available upon reasonable request pending application and approval by the principal investigator.

## Funding

This project was funded by Vitamin Angel Alliance Inc. AM, AP, and VS from Vitamin Angel Alliance Inc provided technical guidance for study design and collection of data. CS received a Canadian Institutes of Health Research (CIHR) Canada Graduate Scholarship-Master’s (CGS-M), CIHR CGS-M provided no study involvement. JML is supported by an Ontario Women’s Health Scholars award, funded by the Ontario Ministry of Health and Ministry of Long-Term Care, and an Ontario Graduate Scholarship, all of which provided no study involvement. CDK is a Michael Smith Foundation for Health Research Scholar and a CIHR Canada Research Chair in Micronutrients and Human Health.

## Conflict of interest

AM, AP., and VS. report a relationship with Vitamin Angel Alliance Inc that includes employment. CDK. reports a relationship with Helen Keller International that includes consulting or advisory, nonfinancial support, and travel reimbursement. All other authors report no conflicts of interest.
