# Supplementary material for: Assessing the adherence and acceptability to iron and folic acid compared with multiple micronutrient supplements during pregnancy: a cluster-randomized noninferiority trial in Cambodia
Source: Am J Clin Nutr. 2025 May 5;122(1):166–73. doi: 10.1016/j.ajcnut.2025.04.033 (PMC12308091; doi:10.1016/j.ajcnut.2025.04.033)
Supplement: Supplementary file 1 [file mmc1.docx]

**Supplemental Online Content**

**Supplemental Table 1:** Adherence and ANC attendance outcomes

**Supplemental Table 2:** Sensitivity analysis

Supplemental Table 1: Adherence and ANC attendance outcomes on the first and second home visit^1^

|  | IFA-90  *n*=477  (30-day) | IFA-90  *n*=459  (90-day) | MMS-180 *n*=444  (90-day) | MMS-90 *n*=435  (90-day) |
| --- | --- | --- | --- | --- |
|  |  |  |  |  |
| Adherence rate  Unadjusted rate,  mean (95% CI) | 89%  (86, 91) | 91%  (90, 92) | 94%  (93, 95) | 94%  (93, 95) |
| Adjusted rate (model), predicted marginal mean (95% CI) | −  ­­ | 91%  (89, 92) ^a^ | 94%  (92, 95) ^b^ | 94%  (93, 96) ^b^ |
| ANC visit attendance^3^ |  |  |  |  |
| Unadjusted mean (95% CI)  Adjusted model, predicted marginal mean (95% CI) | −  − | 2.6  (2.5, 2.6)  2.6  (2.4, 2.7) ^a^ | 2.7  (2.7, 2.8)  2.7  (2.6, 2.9) ^a^ | 2.8  (2.7, 2.9)  2.8  (2.6, 2.9)^a^ |

Abbreviations: ANC, antenatal care; CI, confidence interval; IFA, iron and folic acid; IFA-90, IFA for 90 d; MMS, multiple micronutrient supplements; MMS-180, MMS for 180 d via 1 180-tablet bottle; MMS-90, MMS for 180 d via 2 90-tablet bottles.

^1^All values are mean or marginal means (95% CI). ­­­

^2^A generalized linear mixed-effects model was used to predict marginal mean (95% CI) for each group accounting for health center clusters. Marginal mean values with the same superscript letter in a row are not statistically different across intervention groups (*P*<0.05; Bonferroni-adjusted for multiple comparisons).

Supplemental Table 2: Sensitivity analysis^1^

|  | IFA-90  *n*=459  (90-day) | MMS-180 *n*=453  (180-day) | MMS-90 *n*=443  (180-day) | MMS-180 vs. IFA-90 comparison^2^ | |
| --- | --- | --- | --- | --- | --- |
|  |  |  |  | Mean difference (95% CI) | *P* value |
| Adjusted rate (model), predicted marginal mean (95% CI) | 91%  (89, 92)^a^ | 94%  (93, 96)^b^ | 95%  (93, 96)^b^ | 3.7 (1.7, 5.9) | *P*<0.001 |

Abbreviations: CI, confidence interval; IFA, iron and folic acid; IFA-90, IFA for 90 d; MMS, multiple micronutrient supplements; MMS-180, MMS for 180 d via 1 180-tablet bottle; MMS-90, MMS for 180 d via 2 90-tablet bottles.

^1^All values are mean or marginal means (95% CI). ­­­

^2^A generalized linear mixed-effects model was used to predict marginal mean (95% CI) for each group accounting for health center clusters, education, previous miscarriage, distance from HC, ID poor standing, age, first pregnancy, wealth quintile and gestational age. Marginal mean values with the same superscript letter in a row are not statistically different across intervention groups (*P*<0.05; Bonferroni-adjusted for multiple comparisons).
